# Innovations in service learning: a novel program for community service at NYU School of Medicine

**DOI:** 10.3402/meo.v20.28379

**Published:** 2015-09-18

**Authors:** Nola Seta Herlihy, Christina Brown

**Affiliations:** Office of Student Affairs, NYU School of Medicine, New York, NY, USA

**Keywords:** medical education, medical curriculum, community service, community care

## Abstract

**Problem:**

As NYU medical students, the authors determined that there was no structured form of service learning in their curriculum. They sought to establish a service program that recognizes students for their dedication to community service in both the NYU and NYC communities.

**Approach:**

In 2012, with the support of the Office of Student Affairs (OSA), the authors created the NYU School of Medicine Community Service Program (CSP). The program tracks and verifies students’ participation in service projects. It sets a goal for students to complete 100 service hours through at least five unique service initiatives. Two reflective essays at the completion of pre-clinical and core clerkship curricula challenge students to express how their service experiences will inform their future careers in medicine. The authors developed an innovative online portal for students to track their service involvement and allow the committee to easily approve hours. They created the Community Service Committee, made up of two representatives from each class year, to be in charge of regulating the program together with the OSA.

**Outcomes:**

The class of 2015 is the first class to participate; thus far, 13 students have met program requirements. In the classes of 2016 and 2017, 20 and 41 students, respectively, are expected to receive the award. Total participation has significantly increased in successive class years.

**Next steps:**

The authors seek to gather data on CSP participants’ changing perspectives and hope the program can serve as a model for other schools to build service learning into their curricula.

Service learning is a fundamental component of medical education and training for future physicians. Involvement in student service organizations has been shown to strengthen leadership skills and empathy (1, 2). Previous studies have demonstrated that participation in service-learning initiatives is associated with students reporting greater commitment to continued service and working in underserved communities (3, 4).

As NYU medical students, we determined that there was no structured form of service learning in our curriculum. There were multiple opportunities to participate in community service through student organizations, and we hypothesized that there was already high participation in service events. However, there was no intentional service-learning programing to promote reflection and solidification of the important lessons to be gained from this service engagement. Also, NYU had no forum for recognizing and encouraging longitudinal involvement in community service. Furthermore, few programs have been designed nationwide to engage students in community service in a longitudinal manner throughout their 4 years of medical school. Those programs that do exist are often offered through an elective or selective, thus exposing students to the value of service for just a short time period (5, 6). Other programs are structured around a student-run clinic, in which service is limited to providing care in the role of a physician (7).

We, therefore, sought to design and establish an innovative service-learning program that recognizes students for their dedication to community service in both the NYU and New York City communities, while requiring student engagement in structured reflection during both pre-clinical and clinical years. Our goals were fourfold: to encourage students to participate in a wide array of service activities, to develop a way for students to track their cumulative service involvement throughout medical school and receive timely feedback and validation, to promote direct learning from service involvement through active reflection, and to create a mechanism for NYU to formally recognize students demonstrating extensive and longitudinal community service engagement.

## Approach

In the spring of 2012, we partnered with the Office of Student Affairs (OSA) to create the NYU School of Medicine Community Service Program (CSP). To encourage both a depth and breadth of service with a rigorous, yet attainable goal, program requirements are for students to complete at least 100 service hours through at least five unique service initiatives; no more than 50 h from one distinct service activity can count toward program completion. NYU has a three semester pre-clinical curriculum. As most extra-curricular involvement is performed during this time in medical education, 100 h was determined to be a reasonable expectation.

### Service activity monitoring

**Fig. 1:**
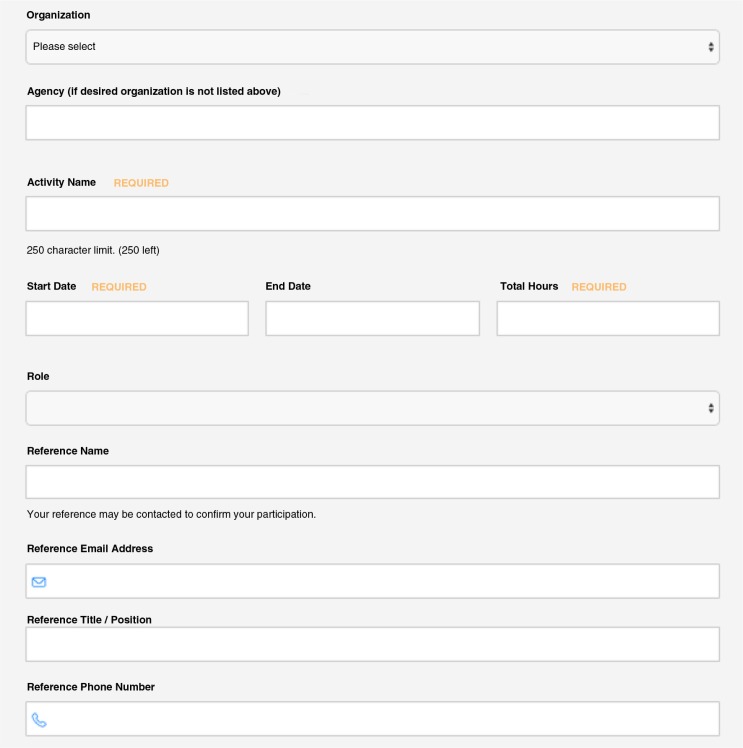
Sample service entry form for NYU School of Medicine Community Service Program.

To address our goal of tracking and monitoring service involvement, we created an online portal through OrgSync, a software company. The portal is a venue for centralizing program guidelines and updates as well as submitting service entry forms (Fig. 1) and reflections. Once students submit an entry, it is added to the community service committee's queue for either approval or rejection. If approved, entries are added to each student's cumulative service involvement log. Entries are typically denied for two reasons: providing incomplete information and requesting service hours for activities not meeting CSP service guidelines. If the former, students may make modifications and resubmit. This approval process allows for both feedback and verification of all service activities on a rolling basis. Other key features that allow for monitoring program participation metrics include the ability to export individual student, single class or total program participation summaries to excel and PDF formats.

### Student leadership

As the NYU CSP was a student-led initiative, one priority was to maintain student involvement in program leadership and guarantee the longevity of the program. To accomplish this goal, we created the Community Service Committee, comprised of two representatives from each class year, which is responsible for regulating the program and acting as liaisons between the student body and the OSA. Committee members from each class are in charge of checking entries to ensure that they are complete and adhere to program definitions of service. Representatives have additional responsibilities according to class year, from organizing the NYU Day of Service to planning program completion.

The creation of the Community Service Committee was vital to program success as it took the burden away from the OSA to maintain daily monitoring and correspondence regarding individual students’ service hour entries. Meanwhile, the committee simultaneously created a valuable leadership role for students interested in and dedicated to promoting service-centered community values at NYU.

As student representatives handle quality assurance on a daily basis, program operation also relies on close communication between the committee and the OSA. We addressed this requirement by establishing a clear protocol for problem resolution. Student questions are first directed to the two class representatives and resolved at the class level if straightforward. Any issues not clearly described by existing published program guidelines are then discussed between all members of the committee to determine if a similar issue has been settled in the past. If program guidelines do not address the issue, then the committee defers to the OSA to make a final policy determination and updates published guidelines accordingly. This system allows service approval policies to remain consistent across all class years, while allowing for continual program evolution.

### Program evolution

Shortly after program implementation, we recognized that not all service activities are of the same caliber and established three ‘categories of service’ to address this issue. Traditional service activities in which students directly serve the community (i.e., preparing meals at a soup kitchen) were renamed ‘Direct External’. Activities that benefit our school community such as serving on lunch panels for the office of admissions or teaching younger students how to draw blood were renamed ‘Direct Internal’. Finally, time spent planning a service event was dubbed ‘Administrative’. The latter two of these categories were each capped at 20% of total program service hours, so that at least 60% of service involvement is required to be community-centered ‘Direct External’.

We quickly determined that student ‘estimation’ or recollection of past service tended to be more inaccurate than a rolling reporting system. Therefore, policies were established requiring students to enter hours in a timely fashion via semester deadlines. Longitudinal service activities (i.e., repeated weekly commitments) were required to be entered on a week-by-week basis without a ‘grouped’ entry encompassing an entire semester of involvement. Proof of participation became required for all off-campus service activities, either via written confirmation or a photo of the event.

Finally, we discovered the importance of careful presentation of the program to the entering class. In the infancy of NYU's CSP, it was introduced to only interested students in an optional after-school meeting. In the third year of the program, the Community Service Committee arranged to make a presentation at a mandatory Student Orientation event, thereby reaching the entire entering class and emphasizing the official character of the program. Participation boosted from 42 to 41 students in the first two participating classes to 88 students with this change in student recruitment.

### Service learning

One of the primary goals of the NYU CSP was to provide a structured framework to encourage students to participate in service and to enrich their experience and professional growth from these activities through reflection (Table 1). This service-learning component is what distinguishes the NYU structure from a superficial community service hour log sheet. Two junctions in the curriculum stand out as particularly significant: completion of pre-clinical years and completion of core rotations. At the completion of pre-clinical work, students are most actively involved in service and service leadership roles and are most prepared to reflect on their direct volunteer engagement in the community. At this point, we ask students:

Explain how service has contributed to your medical education and helped shape the physician you want to be.

**Table 1:** Sample passages from NYU School of Medicine student reflections for Community Service Program

Prompt	Response
Explain how service has contributed to your medical education and helped shape the physician you want to be	In the middle school classroom, I found myself stuttering on medical jargon, ultimately learning to replace it with kid-friendly words, and using tangible analogies to explain difficult concepts. Teaching children about the cardiovascular system allowed me to improve my ability to explain intricate medical concepts to patients.
	Meeting individuals from a variety of ethnicities, religions, ages, and socio-economic backgrounds, with very personal stories, is invaluable during medical school. It highlights the importance of the doctor–patient relationship, emphasizing how every individual is unique and how vital it is to appreciate each perspective.
How has your year of clinical rotations affected your perception of service and its place in medicine?	I think service can also be expanded toward our peers and colleagues. Core clerkship year was stressful for everyone, and we all needed each other to pick us up and put us back together when we fell apart. Providing that kind of emotional and educational support counts as service because it isn't something we always do.
	My peers and I happen to have chosen a field that revolves around providing service to others. It is extremely important to not lose sight of this. Yes, medicine will one day be our jobs and our careers, and at times it may feel repetitive or rote. But, we must remember that at its core we are providing a service, and this means that our last patient of the day deserves as much attention, patience, and care as our first.

During clerkship year, many students have limited direct service involvement. However, all students are engaged in direct patient care on a daily basis. Stemming from these experiences, students tend to change their perspectives on their role as physicians and define the type of physician they wish to be. At this point the CSP asks students:

How has your year of clinical rotations affected your perception of service and its place in medicine?

Through these two prompts, students are tasked with sufficient depth of reflection, requiring introspection that mirrored their own growth as future physicians. These essays are read by students’ academic advisors and incorporated into the medical student performance evaluation (MSPE, or ‘dean's letter’) when appropriate.

### Student recognition

Our final aim was to provide formal recognition for students who complete program requirements demonstrating their dedication to service. While many medical students participate in some community service, the authors and the OSA agree that a process needed to be developed to support and recognize students with an exceptional commitment to serving others. Therefore, in partnership with the OSA, the CSP provides an official certificate of completion that is conferred at a ceremony in the spring of the academic year.

Students are required to submit a final application for program completion which includes total service hours completed, number of hours in each category of service, a list with descriptions of the five minimum service activities and a list of any service leadership roles held. This application enables efficient information processing and corroboration with approved service logs.

The deadline for program completion is April of MS3 year, which enables this achievement to be reflected in residency applications. However, fourth-year students who need additional time for program completion are able to complete the program in a second wave of approvals the following year.

## Outcomes

**Fig. 2:**
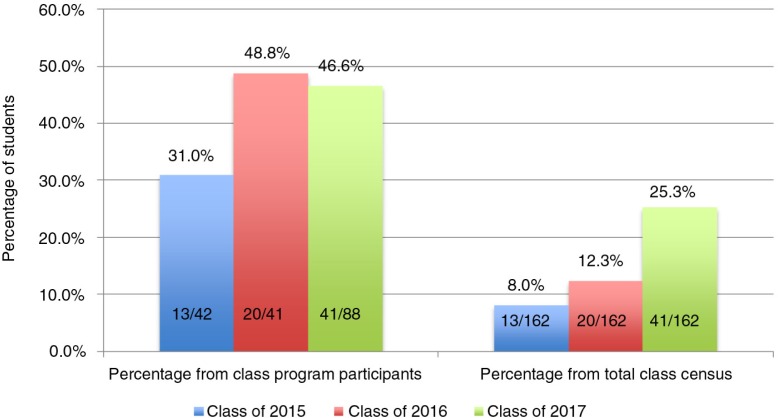
Percentage of NYU School of Medicine students on track for Community Service Program (CSP) completion, both as a fraction of total class census and among CSP participants by class year for the Classes of 2015–2017.

In the 3 years since program implementation, NYU students have recorded 14,781 h of community service. The Class of 2015 is the first class to participate in the program; and thus far, 13 students have met program requirements. In the Classes of 2016 and 2017, 20 and 41 students, respectively, are expected to receive the award at the end of the MS3 year, an estimate based on the suggestion that students complete 70% of their service hours in the pre-clinical years (Fig. 2). With changes in program orientation and recruitment, total participation has increased from 42 and 41 students in Class of 2015 and Class of 2016, respectively, to 88 and 74 students from the Class of 2017 and Class of 2018. Despite boosted numbers of participating students, average hours of community service are not significantly different (*p*=0.84) across classes: Class of 2015 with 76.2 h, Class of 2016 with 75.3, and Class of 2017 with 71.5 h. In addition, the Class of 2018 has an average of 29.7 h with two of three pre-clinical semesters and their first year summer to continue service efforts.

## Next steps

The CSP has grown and evolved rapidly over the past 3 years; we project that it will continue to improve as we introduce new ideas and inspire more students to participate. With increased participation, we hope to create two tiers of achievement (e.g., a separate distinction for those who complete over 150 h). To recognize those students who have demonstrated the most significant commitment to service, we are creating a ‘Community Service Award’ to be given out at the graduation awards ceremony. Finally, it is our hypothesis that participating in our service-learning program is positively affecting students’ leadership skills, attitudes toward working with underserved populations, and professional development. In the future, we seek to obtain further data from students before and after participating in the CSP to support this hypothesis. Medicine is at its core a service profession, and there are many lessons to be learned in the context of service about compassion, humanity, and humility. We hope that our program can serve as a model for other schools to build service learning into their curricula as well.
